# Determining the Maternal and Fetal Cellular Immunologic Contributions in Preterm Deliveries With Clinical or Subclinical Chorioamnionitis

**DOI:** 10.1155/S1064744997000471

**Published:** 1997

**Authors:** M. F. McNamara, T. Wallis, F. Qureshi, S. M. Jacques, B. Gonik

**Affiliations:** 1 Department of Obstetrics and Gynecology Wayne State University School of Medicine Detroit MI USA; 2 Department of Pathology Wayne State University School of Medicine Detroit MI USA; 3 Department of Obstetrics and Gynecology NMC Balboa 36400 Bob Wilson Drive San Diego CA 92134 USA

## Abstract

*Objective:* Our purpose was to determine the maternal and fetal polymorphonuclear contributions to preterm histologic chorioamnionitis and whether this response differs in clinical chorioamnionitis when compared to cases without clinical chorioamnionitis.

*Methods:* Paraffin placenta blocks from 19 preterm deliveries with histologic chorioamnionitis, 9 with clinical chorioamnionitis and 10 without clinical chorioamnionitis, were identified. Only placentas from male fetuses were used. Cytospin slides were generated from tissue specimens for fluorescent in situ hybridization (FISH) and labeled with X and Y chromosome probes. Under fluorescent microscopy, polymorphonuclear cells (PMNs) were identified as having two XX signals (maternal) or a single X and Y pair (fetal).

*Results:* Maternal PMNs comprised 89% and 91% of the cellular response in the groups with and without clinical chorioaminionitis, respectively. This difference in the two groups was not statistically significant.

*Conclusions:* The dominant contribution of PMNs seen in preterm severe histologic chorioamnionitis is maternal in origin. This response is similar in the presence or absence of clinical chorioamnionitis.


Infectious Diseases in Obstetrics and Gynecology 5:273–279 (1997)
© 1998 Wiley-Liss, Inc.

### Determining the Maternal and Fetal Cellular Immunologic Contributions in Preterm Deliveries With Clinical or Subclinical Chorioamnionitis

M.F. McNamara,[1*] T. Wallis,[2] F. Qureshi,[2] S.M. Jacques,[2] and B. Gonik[1]

[1]*Department of Obstetrics and Gynecology, Wayne State University School of Medicine, Detroit, MI*
[2]*Department of Pathology, Wayne State University School of Medicine, Detroit, MI*

ABSTRACT

*Objective:* Our purpose was to determine the maternal and fetal polymorphonuclear contributions to preterm histologic chorioamnionitis and whether this response differs in clinical chorioamnionitis when compared to cases without clinical chorioamnionitis.

*Methods:* Paraffin placenta blocks from 19 preterm deliveries with histologic chorioamnionitis, 9 with clinical chorioamnionitis and 10 without clinical chorioamnionitis, were identified. Only placentas from male fetuses were used. Cytospin slides were generated from tissue specimens for fluorescent in situ hybridization (FISH) and labeled with X and Y chromosome probes. Under fluorescent microscopy, polymorphonuclear cells (PMNs) were identified as having two XX signals (maternal) or a single X and Y pair (fetal).

*Results:* Maternal PMNs comprised 89% and 91% of the cellular response in the groups with and without clinical chorioaminionitis, respectively. This difference in the two groups was not statistically significant.

*Conclusions:* The dominant contribution of PMNs seen in preterm severe histologic chorioamnionitis is maternal in origin. This response is similar in the presence or absence of clinical chorioamnionitis. Infect. Dis. Obstet. Gynecol. 5:273–279, 1997. © 1998 Wiley-Liss, Inc.

KEY WORDS
chorioamnionitis; fluorescent *in situ* hybridization; prematurity

Prematurity is a leading cause of neonatal morbidity and mortality,[1] with infection playing a major role in preterm labor and delivery.[2] In this regard, numerous investigators have found an increased incidence of histologic evidence of chorioamnionitis in the preterm delivered placenta.[1–5] Guzick and Winn[1] found on examination of 2,774 placentas that the rate of histologic chorioamnionitis was 32.8% and 10% in preterm and term placentas, respectively. The majority of the preterm cases with chorioamnionitis are subclinical, with the mother exhibiting no overt features of infection. Conversely, histologic chorioamnionitis is less commonly associated with maternal fever, tachycardia, uterine tenderness, and other typical signs of clinical infection.[6] Microscopic examination of the placenta shows a similar degree of polymorphonuclear (PMN) leukocyte infiltration of the chorioamnion in both circumstances, making distinction between the two scenarios difficult on pathologic grounds.

The above dichotomy leads to speculation as to why such a similar histologic picture is present in two distinctly different clinical situations. Perhaps, in the continuum of this disease process, the inflammatory response precedes clinical evidence of

*Correspondence to: Michael F. McNamara, Department of Obstetrics and Gynecology, NMC Balboa, 36400 Bob Wilson Drive, San Diego, CA 92134.

Received 8 November 1996

Accepted 23 June 1997

disease; however, this seems unlikely given the similarity of histopathologic findings. Another possibility is that the origin of this inflammatory response is different, with the inflammatory response being primarily maternal in the clinical cases and fetal in the subclinical cases. Acute chorioamnionitis frequently shows histopathological evidence of both maternal and fetal response to maternal infection. The earliest response is considered to be maternal and is manifested by PMNs migrating through decidual (maternal) blood vessels into the decidua, often adjacent to the point of membrane rupture or to the dilating cervix. Maternal PMNs also migrate early from the intervillous space into the subchorionic fibrin and membranes toward the amniotic cavity. Fetal reaction may also be observed but is generally delayed and less marked than the maternal response. The fetal response manifests as migration of PMNs through blood vessels of the chorionic plate and umbilical cord, again toward the amniotic cavity.[7] The relative proportions of maternal and fetal PMNs in clinical and subclinical chorioamnionitis have never been exactingly quantified. This is due, in part, to the previous lack of available technology to differentiate between fetal and maternal inflammatory cells. Recently, Redline and Patterson[8] have shown the ability of fluorescent *in situ* hybridization (FISH), using X and Y chromosome probes to determine the origin of the inflammatory infiltrate in chronic villitis.

The basis of the present study was to utilize FISH, with the X and Y chromosome probes, to examine the origin of the inflammatory response seen in preterm chorioamnionitis, and to determine if it was different in those with and without clinical symptomatology.

### SUBJECTS AND METHODS

### Specimen Collection

Placental pathology records were reviewed from 1992 through 1995 for cases of histologic chorioamnionitis in preterm deliveries between 25 and 34 weeks gestational age (n = 164). Chorioamnionitis was defined as the presence of PMN infiltration of the chorioamnion. Available hospital records associated with these pathology reports were accessed and reviewed for demographic and clinical information. Only pregnancies with the delivery of a male infant were studied, so as to use the presence or absence of the Y chromosome to distinguish between maternal and fetal PMNs (n = 87). Two groups were identified based on the clinical presentation as reported in the chart: clinically overt and subclinical chorioamnionitis. From each group, 10 cases were randomly chosen for evaluation. Two cases of clinical chorioamnionitis with the delivery of a female infant were also included (n = 2) in the blinded evaluation. The cases were reviewed to ensure the presence of quantifiable PMNs by FISH techniques. All cases examined by FISH showed full thickness infiltration by PMNs of the chorioamnion and vasculitis, considered evidence of maternal and fetal responses, respectively. Clinically overt cases were identified as those presenting with maternal fever, uterine tenderness, and maternal or fetal tachycardia. All of these cases were treated with intravenous broad spectrum antimicrobial therapy directed against intraamniotic infection. Subclinical cases lacked these intrapartum findings for infection, and received only group B streptococcus prophylaxis in labor due to their preterm status. One case in the clinical group was later excluded due to the antepartum treatment of syphilis.

### Cytospin Preparation

Formalin-fixed and paraffin-embedded placenta samples of the 19 study cases were obtained. Each sample, obtained from the central portion of the placenta, was blindly reviewed by both pathologists (F.Q. and S.M.J.) to confirm the diagnosis of histologic chorioamnionitis (Fig. 1). Full thickness, 36 μm tissue sections were cut from selected blocks, deparaffinized in xylene, and then rehydrated in decreasing concentrations of alcohol. A protease solution was used for tissue digestion. The solutions were then poured through a 50 μm filter separating the tissue from individual cells and refrigerated. Gentle centrifugation yielded a representative cell suspension for cytospin slide preparation. The cytospin slides were generated using two drops of the cell suspension and one drop of albumin. Four slides of each suspension were made.

### FISH

The cytospin slides were air dried. One slide from each specimen was chosen for FISH. FISH probe and detection kits were obtained from Oncor, Inc.

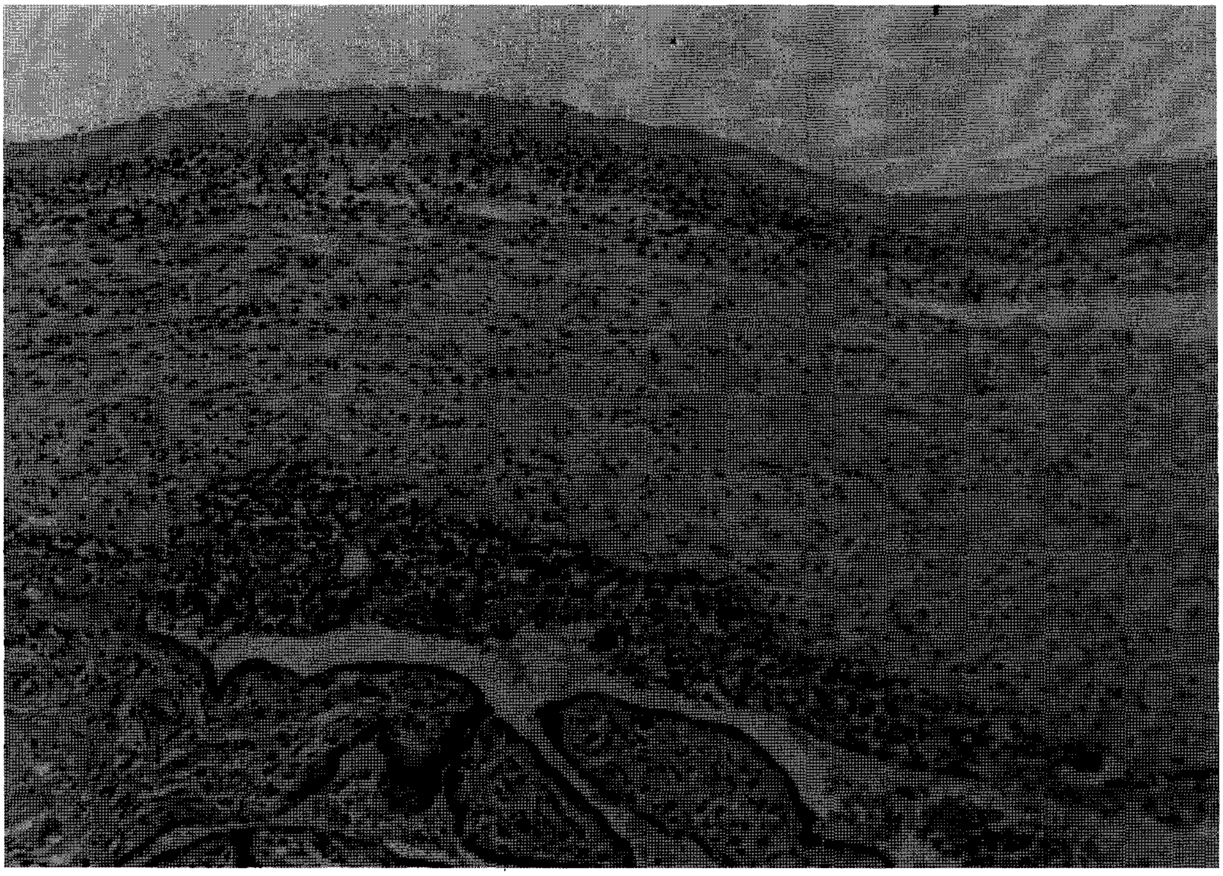

Fig. 1. Placenta section showing chorioamnionitis.

(Gaithersburg, MD). Satellite probes for X and Y chromosomes were placed on the cytospin slide and incubated on a 90°C heating plate for 12 min for protein denaturation. The slides were then placed in a prewarmed humidified chamber at 37°C for hybridization and incubated for 16 h. A dual-color detection agent, consisting of rhodamine-labeled anti-digoxigenin and fluorescein-labeled avidin, was added to each slide and incubated at 37°C for 5 min. Amplification of the X and Y probes was performed, followed by counterstaining with a DAPI/antifade preparation. A cytospin slide from each specimen also underwent staining with hematoxylin-eosin (H&E), and the slides were concomitantly reviewed by one of the coinvestigators to evaluate the morphology of the cells in the cytospin specimen. PMNs were easily distinguished morphologically from decidual and trophoblastic cells (Fig. 2).

Fluorescent microscopy was utilized to evaluate the slides. Alternating filters, 100 PMNs were identified and evaluated for presence of fluorescent probes. Each PMN was evaluated for the presence of two fluorescent probes within the cell nucleus. The X probe appeared green, while the Y probe appeared red. The presence of two X probes signified a PMN of maternal origin (Fig. 3). A single X and Y pair signified fetal origin of the PMN (Fig. 4).

### Statistical Analysis

Demographic and clinical variables between the two study groups were compared using the Student's t-test. Means and standard deviations (SD) for the maternal and fetal PMN contributions for the clinical and subclinical cases were calculated. $P < 0.05$ was considered significant.

### RESULTS

Blinded examination of the 21 cytospin FISH preparations was undertaken. This included a clinical chorioamnionitis group (n = 9), subclinical

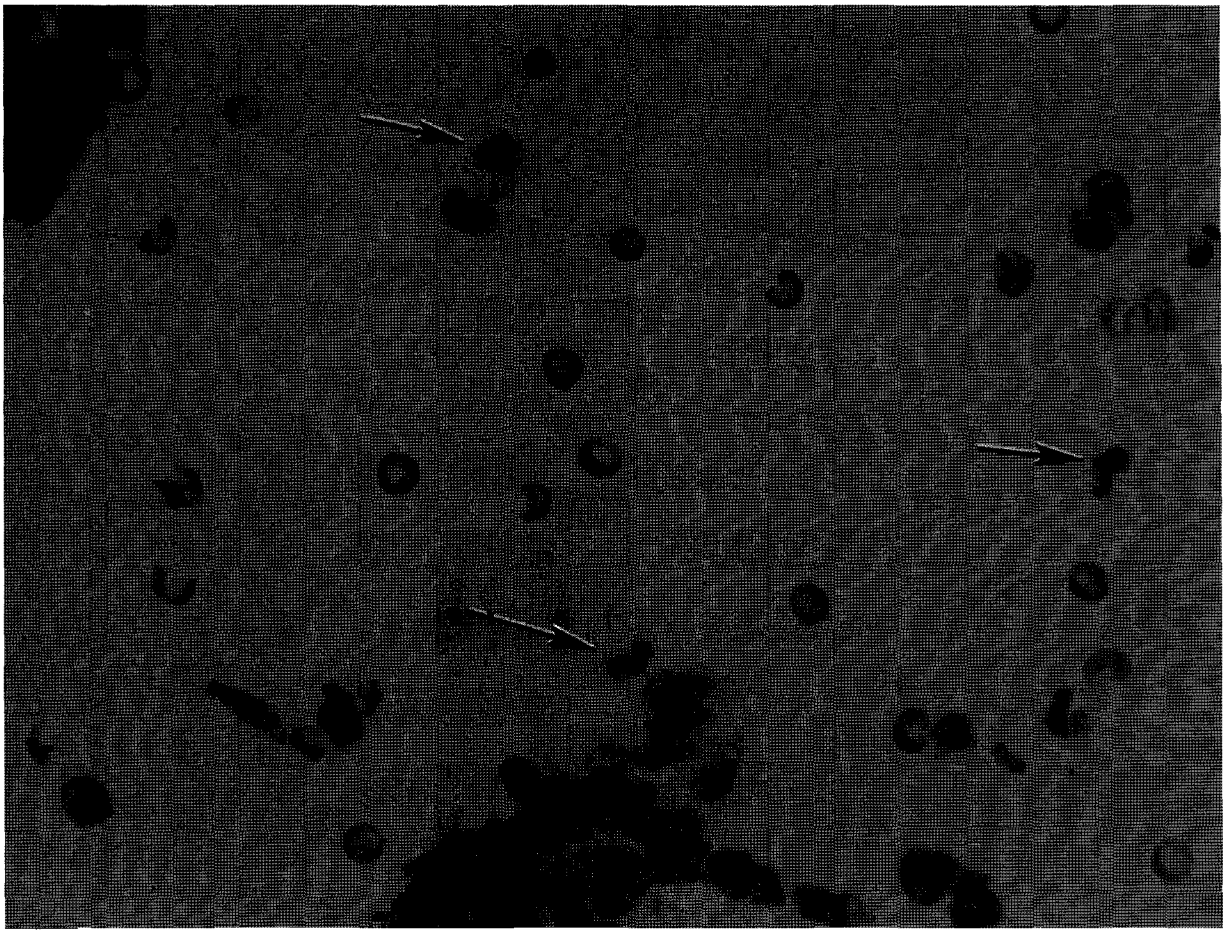

Fig. 2. H&E staining of cytospin specimen, arrows identifying PMNs.

group (n = 10), and the 2 female infants with clinical chorioamnionitis. The placental specimens from the clinical chorioamnionitis group demonstrated a mean maternal (XX) PMN count of 88.33 (SD 5.94). The placental specimens from the group without clinical chorioamnionitis had a mean maternal PMN count of 91.4 (SD 3.13). The two placental specimens from female infants with choriamnionitis revealed only XX PMNs. Using the Student's t-test, there was no significant difference in the two study groups with regard to the ratio of the maternal and fetal PMN responses ($P = 0.17$). Table 1 lists demographic and clinical characteristics of the clinical and subclinical chorioamnionitis groups. There was no significant difference between the two groups with respect to gestational age ($P = 0.056$). There was a significant difference in two areas. The first was birth weight, with a mean weight of 1,365.89 g in the clinical group and 1,906.4 g in the subclinical group ($P = 0.021$). The second factor was the length of membrane rupture prior to delivery. The clinical group's mean was 128.44 h, while the mean in the subclinical group was 17.7 h ($P = 0.005$). There was no significant difference in maternal age or parity between the two groups.

### DISCUSSION

Numerous studies have linked infection and premature delivery. Studies reviewing preterm delivery and placental pathology reveal a significant incidence of histologic chorioamnionitis.[2,9] The placental findings are similar despite differences in the clinical presentation. PMNs are a sign of acute infection in the placenta. The origin of these PMNs, whether maternal or fetal, has not been exactingly quantified in the past. It was though that in the two distinct clinical scenarios, the origins of the PMNs would be different. In our evaluated cases, we did not find a significant difference. The groups did display two significant differences: length of ruptured membranes and birth weight.

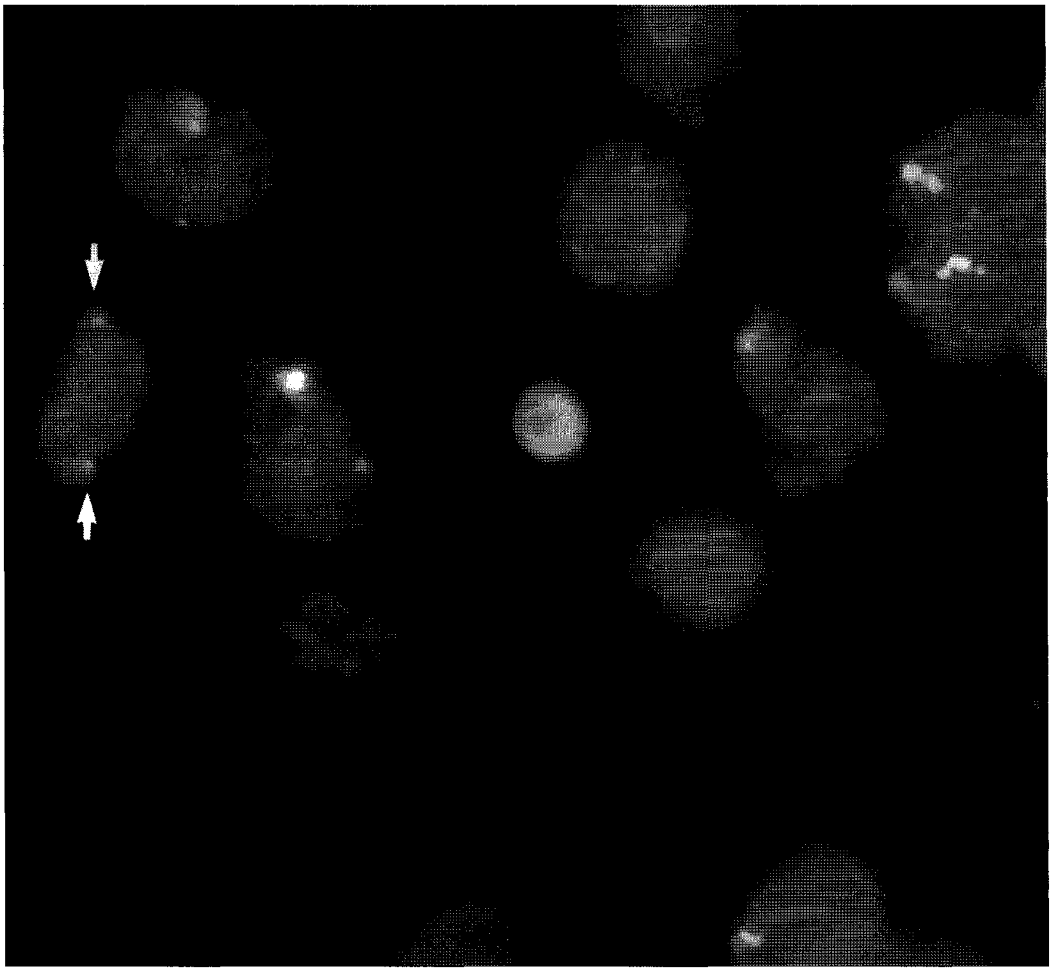

Fig. 3. FISH of a maternal PMN with two X signals (arrows).

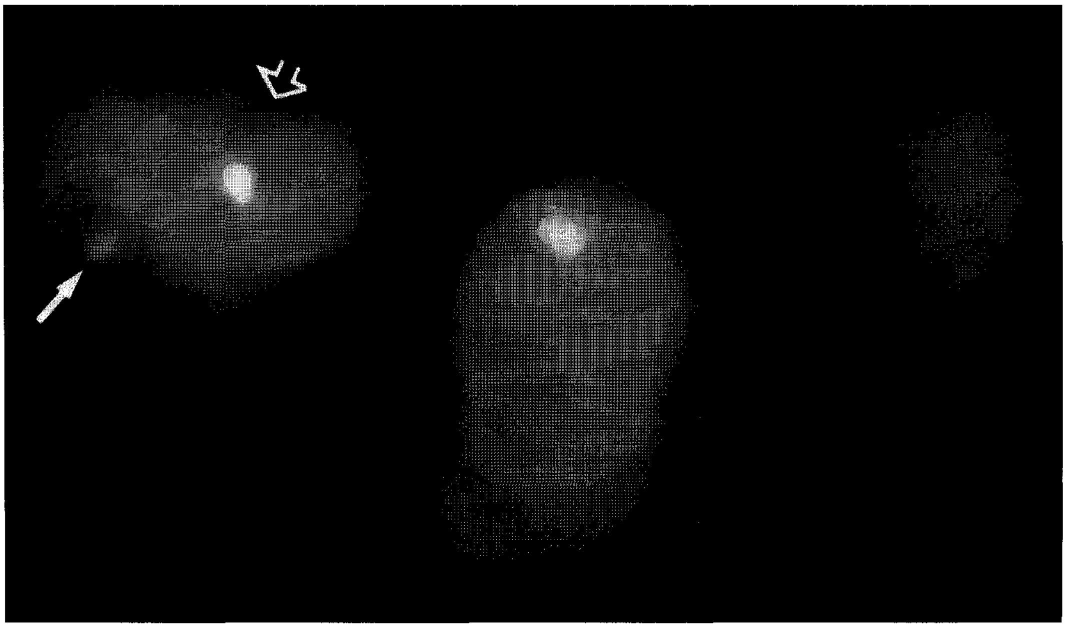

Fig. 4. FISH of a fetal PMN with an X (solid arrow) and a Y (open arrow) signal.

TABLE 1. Maternal and fetal characteristics

| | Clinical chorio-amnionitis (n = 9) | Subclinical chorio-amnionitis (n = 10) | P |
|---|---|---|---|
| Parity | Mean 2.78<br>SD 2.59 | Mean 2.20<br>SD 1.55 | 0.557 |
| Estimated gestational age weeks | Mean 29.4<br>SD 2.79 | Mean 31.54<br>SD 1.67 | 0.056 |
| Birth weight (g) | Mean 1,365.89<br>SD 575.33 | Mean 1,906.4<br>SD 336.51 | 0.021 |
| Ruptured membranes to delivery (h) | Mean 128.44<br>SD 103.66 | Mean 17.7<br>SD 28.41 | 0.005 |
| Maternal (XX) PMNs (per 100 cells) | Mean 88.33<br>SD 5.94 | Mean 91.4<br>SD 3.13 | 0.17 |
| Fetal (XY) PMNs (per 100 cells) | Mean 11.67<br>SD 5.96 | Mean 8.6<br>SD 3.13 | 0.17 |

The clinical chorioamnionitis group had a longer length of membrane rupture than the subclinical group. This finding is not surprising as the incidence of clinical chorioamnionitis has been shown to increase with the length of membrane rupture. It is interesting that despite the increase in duration of rupture of membranes and presence of clinical chorioamnionitis in this group, there was no significant difference in the ratio of maternal to fetal PMNs. The other area of significant difference was birth weight. This is most likely due to the difference in gestational age between the two groups. The mean birth weight for infants born at 29 weeks during this period was 1,367.5 g (n = 145) and 1,814.3 g (n = 242) at 31 weeks.

FISH permits an opportunity to distinguish maternal from fetal nucleated cells. This is possible in cases where there is a distinct chromosome difference between the mother and the fetus such as aneuploidy or a male fetus. Redline and Patterson[8] described the technique using FISH to study the origin of chronic inflammatory cells in cases of chronic villitis of unknown etiology. Cowles et al.[10] used FISH on cell suspensions of placental tissue of chromosomally normal and abnormal infants and were able to accurately identify the abnormal karyotypes.

Our initial attempts at FISH techniques were using mounted paraffin sections. We encountered difficulties with tissue architecture and cell overlap. The loss of architecture was felt to be secondary to the deparaffination process but similar results were observed when using fresh placental sections. Cell overlap was also a problem despite using 4–5 μm tissue sections. Adequate evaluation of the PMN nuclei and individual fluorescent signals was not possible. Thus, we were unable to quantify the degree of maternal vs. fetal response. The PMN is the predominant nucleated cell seen on histologic section of severe chorioamnionitis. Deparaffination and tissue digestion produced a cell suspension that allowed identification of the PMN and avoided the problem of cell overlap. FISH techniques were possible with good visualization of the X and Y chromosome probes within the individual PMNs.

Our results demonstrate that the fetus does contribute 10–13% of the acute cellular inflammatory response, although the inflammatory response is overwhelmingly maternal. This relationship between the maternal and fetal compartments holds true regardless of the clinical presentation (clinical vs. subclinical chorioamnionitis). However, the possible pathophysiologic mechanism for explaining differences in clinical presentation of histologic chorioamnionitis requires further investigation. Perhaps the source of immune signaling (e.g., interleukin 1 production) is different in clinical and subclinical cases of chorioamnionitis. In the first case, maternally produced cytokines may recruit inflammatory cells at the same time they activate a systemic response. Conversely, in the latter scenario, fetally derived cytokines may recruit local maternal PMNs but are unable to activate a maternal systemic response. The end results in both scenarios are similar contributions by the maternal and fetal compartments, though the origination of the signal for assistance may be different.

### CONCLUSIONS

The preterm fetus does contribute to the acute cellular inflammatory response observed in the placenta, though the maternal response predominates. The presence of clinical chorioamnionitis does not significantly change the magnitude of this response.

### REFERENCES

1. Guzick DS, Winn K: The association of chorioamnionitis with preterm delivery. Obstet Gynecol 65:11–15, 1985.
2. Romero R, Mazor M: Infection and preterm labor. Clin Obstet Gynecol 31:553–584, 1988.
3. Chellam VG, Rushton DI: Chorioamnionitis and funiculitis in the placentas of 200 births weighing less than 2.5 kg. Br J Obstet Gynaecol 92:808–814, 1985.
4. Blanc WA: Pathology of the placenta, membranes and umbilical cord in bacterial, fungal, and viral infections in man. In Kauffman N (ed): Perinatal Diseases. Baltimore: Williams & Wilkins, pp 67–132, 1981.
5. Zlatnik FJ, Gellhaus TM, Benda JA, Koontz FP, Burmeister LF: Histologic chorioamnionitis, microbial infection, and prematurity. Obstet Gynecol 76:355–359, 1990.
6. Gonik B: Amnionitis. In Zuspan FP, Quilligan EJ (eds): Current Therapy in Obstetrics and Gynecology. Philadelphia: W.B. Saunders, pp 208–210, 1994.
7. Gersell DJ, Kraus FT: Diseases of the placenta. In Kurman RI (ed): Blaustein's Pathology of the Female Genital Tract. 4th ed. New York: Springer-Verlag, pp 975–1048, 1994.
8. Redline RW, Patterson P: Villitis of unknown etiology is associated with major infiltration of fetal tissue by maternal inflammatory cells. Am J Pathol 143:473–479, 1993.
9. Mueller-Heubach E, Rubinstein DN, Schwarz SS: Histologic chorioamnionitis and preterm delivery in different patient populations. Obstet Gynecol 75:622–626, 1990.
10. Cowles TA, Elder FFB, Taylor S: Identification of abnormal chromosomal complement in formalin-fixed, paraffin-embedded placental tissue. Prenat Diagn 15: 21–26, 1995.
